# National Meeting Breaks the Mold

**DOI:** 10.1289/ehp.112-a810

**Published:** 2004-10

**Authors:** Julie Wakefield

As the 2004 hurricane season nears its end after an unprecedented run of flooding and other water damage, attention is turning once again to the health effects of toxic mold infestation. Exposure to mold in residential, public, and commercial buildings is thought to have caused health problems ranging from bleeding lungs to hair loss—even to death. But debate continues over many key questions, such how to best treat exposed individuals. In an effort to push through questions that still constrain the field, participants at the June 2004 National Meeting on Mold-Related Health Effects: Clinical, Remediation Worker Protection, and Biomedical Research Issues established a consensus on mold-related health effects and discussed clinical recommendations and a future research agenda for the evaluation, diagnosis, treatment, and management of these health problems.

The meeting was aimed at an interdisciplinary cross-section of policy makers, researchers, engineers, advocacy group members, and clinicians. Sponsors included the NIEHS, the Society for Occupational and Environmental Health, the Association of Occupational and Environmental Clinics, the Johns Hopkins Bloomberg School of Public Health, the Urban Public Health Program of Hunter College, the University of Medicine and Dentistry of New Jersey School of Public Health, and the NIH Office of Rare Diseases.

## A Gamut of Questions

Outstanding research questions on the health effects of mold exposure run a broad gamut. Do airborne fungi produce known or unknown compounds that modulate immunity? Does co-exposure to multiple molds and other allergens occur, and how, and with what effect? Does mold exposure produce neurophysiologic and neurobehavioral abnormalities in children? And how can we best develop registries to chronicle exposures to mold and fungi?

One leading question is whether exposure to high levels of allergens in buildings triggers new-onset allergies. Some clinicians at the meeting had examined individual cases in which mold-contaminated environments appeared to have caused new-onset adult asthma, but population-based research is needed to confirm these findings. Exposure in children seems to cause other respiratory tract disorders besides allergies. These include rhinosinusitis, cognitive and developmental effects, psychological effects, and other nonimmunologic health effects.

To study mold-related health effects, standard assessment tools such as clinical questionnaires for tracking symptoms and effects are needed, as are exposure assessment indicators. To date, questionnaires have proven valuable in assessing population response to abatement. But there are no good, clinically useful biological markers of exposure for nonallergic health outcomes, contended Clifford Mitchell, director of the occupational medicine residency program at the Bloomberg School of Public Health. Participants recommended that diagnostic testing be symptom-based and that exploratory tests for neurobehavioral, neurologic, immunologic, and allergic effects be developed.

Direct and indirect measures should be further developed and validated, said J. David Miller, an industrial researcher in fungal allergens and toxins at Carleton University. Markers of early biological effects might be related to cumulative exposures in moist or contaminated environments. Key questions presented by Michigan State University food scientist James Pestka included whether toxicokinetics and tissue concentrations in animals correlate with *in vitro* effects, and whether airborne exposure data or human tissue levels correlate with thresholds for immune effects in animals. Participants produced a detailed list of research questions, which participants prioritized through a survey after the meeting. The list will be available in a meeting report due out this winter.

## The Public Health Perspective

Without a consensus on specific aspects of mold-related health effects, the primary concern from a public health perspective is that affected people need to be treated and returned to a safe environment. In addition, the mold and the conditions that led to it need to be corrected.

It is difficult to measure people’s exposures to molds, fungi, and their constituents and metabolic products from different sources. For example, many molds and fungi produce mycotoxins that further complicate health effects by acting in a synergistic fashion. Current techniques are limited in their sensitivity and what they can measure, especially given the wide distribution of fungi and complex aspects of growth and metabolism. Factoring in cumulative exposures and all clinically relevant exposures is beyond current capabilities. In general, large integrated samples are needed for accurate exposure assessment.

“The bottom line,” explained Miller, “is that indoor exposure [involves] much more than just fungal material—it’s a lot of stuff.” And from a public health point of view, he said, what’s most important is mitigating and treating the exposure. He acknowledged that the details—for example, knowing the biologically active agent or the specific spore present—may make a difference for policy makers, lawyers, and others.

Once a mold problem is identified, exposed individuals should first be removed from the exposure. Then they should receive treatment depending on symptoms and diagnosis using the tools of evidence-based medicine. Participants noted that treatment for cumulative and toxic exposures should be further researched; doctors do not currently advise prophylactic treatment based on known exposure alone, although symptoms, of course, are treated. The effectiveness of health and remediation interventions also needs probing. It is also important to clearly communicate with exposed populations after interventions to let them know what the exposure means to their health and how to best manage it, Mitchell said.

Yet even after abatement, Mitchell added, some individuals may be symptomatic. “It’s important for everybody to realize there is not a one hundred percent fix for [mold contamination and exposure], and this is a message that needs to go to the clinical world as well as the policy world.”

## Cleanup and Prevention

Many issues remain to be resolved around sampling. Generally, participants agreed that for home abatements, sampling is likely not worth the expense, and it makes more sense financially to just solve the problem. In large buildings (particularly office environments), on the other hand, sampling may be useful to pinpoint the source of exposure, both for legal reasons and for cleanup purposes.

But many remain skeptical of sampling’s ultimate utility. “Sampling does little to add to the diagnosis, management, or correction of the problem,” said Gregg Recer, a research scientist with the New York State Department of Health. And in practice, determining when a building is safe for individuals who experienced mold-related health problems remains a thorny issue. Most experts agree that visual and olfactory inspection by a competent authority with appropriate personal protective equipment before and after abatement is the best strategy.

Work is also needed in developing better guidance for maintenance and remediation workers. There are no standards or requirements for training, said Susan Klitzman, an urban public health professor at Hunter College. Some outfits offer certification, she said, but no hands-on experience—a component that experts at the conference felt was vital.

For now, there is a general consensus that, at a minimum, workers need some type of respiratory protection and gloves. “We can come up with general guidelines, but there’s no one-size-fits-all approach,” Klitzman said. “Professional experience and professional judgment are really paramount here.”

Most of the existing guidance doesn’t cover in sufficient detail other categories of workers who may work in an exposed area on a regular basis, such as maintenance workers, construction workers, teachers, and office workers. Participants will compile new guidance for all groups of workers as a product of the meeting. As Ted Outwater, a public health educator in the NIEHS Division of Extramural Research and Training, concluded, “We’re into this because we view workers as our first line of environmental defense.”

As with many environmental threats, preventing exposure is key for mold; in this case, prevention largely involves correcting moisture problems and housekeeping deficiencies. Participants agreed that remediation goals should include addressing underlying moisture problems, removing or cleaning moldy and damaged materials, protecting workers and occupants, and using containment procedures appropriate for the conditions. Remediation techniques depend on moisture source, condition of the structure and furnishings, building materials, location of mold contamination, presence of additional contaminants, and effects on operations (for example, whether a business will have to be closed down for weeks).

“We have to think very carefully about [performing] outcome studies,” said Mitchell. “At this point we certainly know enough that we have to correct the problem. And figuring out which part of the problem is most important to correct and what that question means for population health is an important research question.” At the same time, he said, we need to understand how those corrective interventions pay off in terms of public health.

## Figures and Tables

**Figure:**
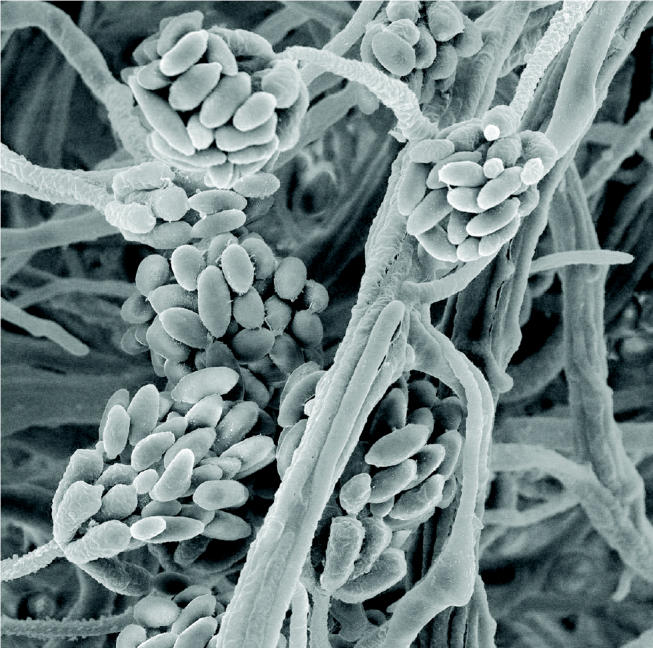
**Public health menace.** *Stachybotrys chartarum hyphae* is just one of many toxic molds whose spores can cause serious adverse health effects when inhaled.

